# Nutrient and physicochemical properties as potential causes of stress in mangroves of the central Red Sea

**DOI:** 10.1371/journal.pone.0261620

**Published:** 2021-12-23

**Authors:** Abdullahi Bala Alhassan, Mohammed Othman Aljahdali

**Affiliations:** 1 Department of Biological Sciences, Faculty of Science, King Abdulaziz University, Jeddah, Saudi Arabia; 2 Department of Biology, Faculty of Life Sciences, Ahmadu Bello University, Zaria, Nigeria; Shandong University, CHINA

## Abstract

Mangrove ecosystems are some of the most productive and important sinks for sediment globally. Recently, there has been an increasing interest in possible causes of stress in mangroves, such as nutrient limitation, high salinity, solar radiation and temperature. We measured different factors casing stress and determined how they influenced oxidative stress and growth biomarkers in six study sites dominated by mangroves; Al Lith, South Jeddah, Dahban, Thuwal, Rabigh and Mastorah. Significant differences (*P* < 0.05) were recorded in water salinities and temperatures, nitrogen and phosphorus content in sediments, and antioxidant enzyme activities in different study sites. The highest salinity (40.75 ‰) and temperature (29.32°C) were recorded in the Rabigh mangrove stand, which corresponds to the lowest dissolved oxygen (5.21 mg/L). Total organic carbon, total nitrogen and total phosphorus in sediment across the study areas were in the order Rabigh>Thuwal>Dahban>Al Lith>South Jeddah>Mastorah. Total nitrogen in mangrove leaves at Rabigh was the highest and about 1.3 times higher than the total nitrogen in South Jeddah mangrove ecosystem, very different from the ratio of total nitrogen in the sediments at Rabigh and South Jeddah mangrove ecosystems. The average values of δ^13^C (-17.60‰) and δ^15^N (2.84‰) in the six mangrove ecosystems, and the highest δ^13^C (-13.62‰) and δ^15^N (4.39‰) at Rabigh in the sediments suggest that nutrient input differed among study sites. Higher nutrient levels at Rabigh mangrove ecosystem were attributed to restricted circulation, camel grazing and land runoff with agricultural waste during seasonal flooding events. However, N limitation and possibly salinity contributed to stress in Al Lith, South Jeddah, Dahban, Thuwal, Rabigh, and Mastorah mangrove ecosystems. Salinity (r = 0.9012) contribute more to stress at Rabigh.

## Introduction

Mangrove ecosystems in marine environments are some of the most productive and important sinks for sediment globally, with shoreline characteristics that provide protection from seasonal flooding [1–5]. In addition, they provide substantially greater gross primary production and sediment carbon and nutrient accumulation relative to environments lacking vegetation cover in marine ecosystems [5–7]. However, the increased capacity of nutrient accumulation in mangrove ecosystems makes this unique ecosystem sensitive to anthropogenic influence within short time period [8, 9]. For instance, variables such as agricultural activities at the catchment of mangroves, urban development, effluent containing sewage, flooding, and sand storms, presumably can lead to an influx of high concentration of nutrients in mangroves, which contributes to nutrient enrichment [10, 11].

Nutrient limitations have been reported among mangroves and other plant communities [12, 13]. However, mangroves can develop physiological strategies such as increasing root biomass and shifts in biomass allocation to withstand nutrient limitations [13]. Nutrient limitation and other factors causing stress such as salinity, solar radiation, and temperature can decrease mangrove height or increase the incidence of dwarfism [14]. Nitrogen and Phosphorus are the elements vastly studied in terms of nutrient enrichment and depletion, with the depletion of N and P reported causing dwarfism in mangroves [15–17]. Indeed, the mangroves of the Caribbean and islands in Southeast Asia and the Red Sea with limited nutrients have been reported to have a dwarf stature [18, 19].

The Red Sea is about 2000 km long and 335 km wide, having mangroves distributed for about 135 km^2^, with some Central Red Sea mangrove ecosystems containing mangroves with dwarf stature [20]. The Indian Ocean serves as one of the major sources of nutrient inputs [21], which causes a gradient of oligotrophication to the north [22], together with an upsurge in salinity as a result of increased evaporation [20, 23]. The central Red Sea is characterized by high temperatures (24.0–33.0°C) and salinity (38.4~39.8 ‰) [24], which are substantially increased by strong evaporation and intense solar radiation. Most mangroves in the central Red Sea are negatively influenced by anthropogenic activities such as urban and industrial development [18] and hydrological events such as seasonal flooding, an influx of land runoff containing agricultural waste, stagnant water, and decreased circulation [19]. However, it is crucial to understand how environmental factors or proxies of stress influence stress in mangroves at the central Red Sea, such as increasing solar radiation, temperature, and evaporation could be triggered by current global warming.

Here we examine carbon and nutrient concentration, nutrient limitation, and environmental factors contributing to stress, and to what degree is the stress has occurred in mangroves at the central Red Sea. Surface sediment (0–20 cm) and *A*. *marina* leaves were sampled from six mangrove stands which were thought to vary in seawater temperature, salinity, pH, dissolved oxygen and sediment nutrients concentrations. The objectives of this study were to determine the order of mangrove ecosystems investigated in terms of nutrient accumulation in sediments, seawater physicochemical parameters and the influence of nutrient and physicochemical parameters on oxidative stress in *Avicennia marina*.

Antioxidant enzymes such as CAT, GST, and SOD are among the basic stress enzymes [25]. They can modulate plants’ growth and physiological activities under environmentally stressed conditions by playing a vital role in removing reactive oxygen species (ROS) such as H_2_O_2_ and O_2_ produced due to stress. This could lead to reducing membrane lipid peroxidation and stabilization of the cell membrane [26, 27]. Antioxidants prevent oxidative damage of cells in plants by scavenging ROS produced during stress conditions; thus, increasing ROS triggers increases antioxidants [19, 25–27]. This process has made it possible and appropriate to use antioxidants as biomarkers of stress [28].

Nitrogen stable isotope values can be employed to get good information on nutrient sources in an aquatic environment [29, 30], while stable carbon isotope provides an understanding of carbon flow contribution from terrestrial and non-terrestrial sources [9]. The nonexistence of a risk assessment by stakeholders and monitoring framework presently for stressors such as nutrient and physicochemical parameters in the central Red Sea mangrove ecosystems, possibly due to anthropogenic activities, is of major concern. Therefore, this study aims to determine the order in which nutrient variation in mangrove sediments and physicochemical parameters such as high temperature and salinity synergistically contribute to stress in *A*. *marina* across six mangrove stands in the central Red Sea. Thus, our findings should support management strategy development by stakeholders and the government to conserve these important ecosystems.

## Materials and methods

### Study area

The Red Sea is composed of about 135 Km^2^ area of mangroves, distributed up to the northern boundary of mangroves at 28.207302°N [24]. The Indian Ocean serves as one of the major sources of nutrient input to the Red Sea, causing a gradient of oligotrophication to the northern part and a rise in salinity due to evaporation [31]. The surface temperature of the Red Sea decreases from the southern part to the north. The central Red Sea is in an arid environment with high temperatures and sparse rainfall; the mean annual (sporadic) rainfall in the Jeddah region is 55 mm, the salinity is relatively high [31], and the nutrient inputs is low. Some mangrove habitats of the Saudi Arabian Red Sea develop as a narrow fringe that could support halophytes located along the shore and adjacent to sand flats which sometimes flood [20].

**Fig 1:**
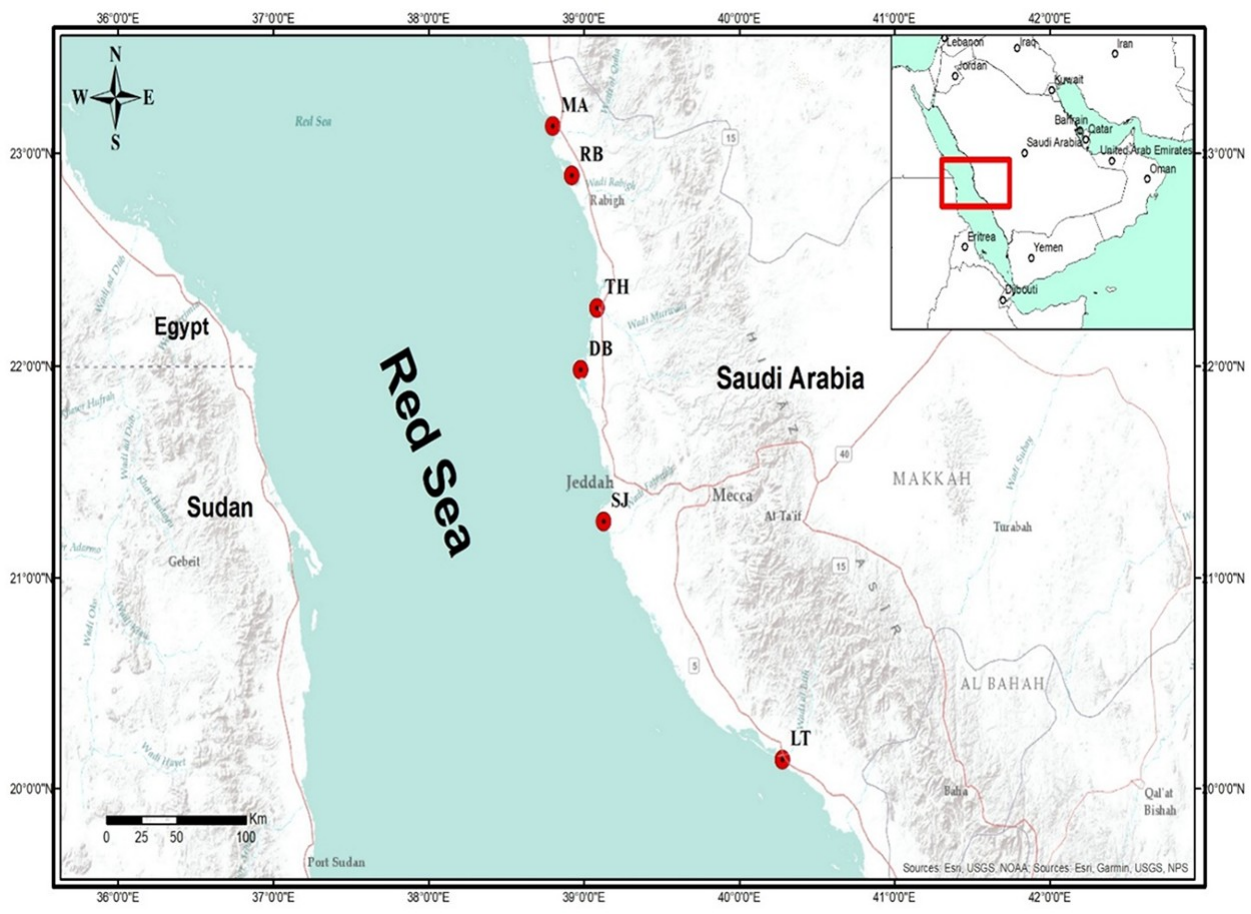
Sampling sites in mangrove stands located on the coast of the central Red Sea, Saudi Arabia. LT—Al Lith, SJ—South Jeddah, DB—Dahaban, TH—Thuwal, RB—Rabigh and MA–Mastorah.

The sampling locations (Fig 1) in this study were chosen based on the spatial distribution of mangroves, differences in the composition of monospecific stands of *Avicennia marina*, abundance and height, anthropogenic activities and anthropogenic sources of nutrients (Table 1). Specifically, six (6) mangrove stands were chosen, which include Al Lith (20°08’~18.70’’N, 40°16’~41.74’’E): Production and extraction of living and non-living resources (aquaculture, fishing and capital dredging). South Jeddah (20°15’~43.92’’N, 40°25’~11.37’’E): Extraction of natural resources (fishing) and seaport in its southern part, power plant, and dredging for maintenance. Dahban (21°59’~05.1’’N, 38°58’~42.9’’E): Recreation-like activities in the catchment and fishing in the mangroves. Thuwal Island (22°16’~36.99’’N, 39°05’~00.34’’E): Tourism or recreation, fishing and desalination plant. Rabigh lagoon (22°53’~51.86’’N, 38°55’~13.25’’E): Large petrochemical complex at the catchment (refinery), receive land runoff passing through agricultural fields, stagnant water due to decreased circulation and livestock activities such as camel grazing.

**Table 1:** Study sites in Saudi Arabia Red Sea with water circulation and residence time characteristics and potential causes and sources of anthropogenic impacts.

	Land use types/Anthropogenic activities/Properties	Distance between study sites	Water circulation and residence time
**Al Lith**	Production and extraction of living and non-living resources (aquaculture, fishing and capital dredging).	Located at the southern part and 148 km from South Jeddah	Large scale coastal circulation, wind induced circulation and reduced water residence time
**South Jeddah**	Extraction of natural resources (fishing) and seaport in its southern part, power plant, and dredging for maintenance	108 km and 148 km from Dhaban and Al Lith respectively	Large scale coastal circulation, wind induced circulation and reduced water residence time
**Dahaban**	Recreation like activities in the catchment and fishing in the mangroves	30 km and 108 km from Thuwal and South Jeddah respectively	Wind induced circulation and reduced water residence time
**Thuwal**	Tourism or recreation, fishing and desalination plant	70 km away from Rabigh	Large scale coastal circulation, wind induced circulation and reduced water residence time
**Rabigh**	Large petrochemical complex at the catchment (refinery), receive land runoff passing through agricultural fields, stagnant water due to decreased circulation and livestock activities such as camel grazing	30 km away from Mastorah	Partial enclosure at the northern end leading to decrease water circulation and increase water residence time
**Mastorah**	No anthropogenic source of nutrients, but ~50 km to the north is an industrial activity	Located at the northern part, 30 km away from Rabigh and about 386 km from Al lith in the southern part	Wind induced circulation and reduced water residence time

Mastorah (23°07’~46.49’’N, 38°47’~56.99’’E): No anthropogenic source of nutrients, but ~50 km to the north is an industrial activity.

### Sample collection and physicochemical parameter of seawater

The field site assess permit (Request number: 23992) was secured from Saudi Border Guard, Ministry of Interior, Saudi Arabia. A total of 90 matured leaves of *A*. *marina* and 90 surface sediment (0–20 cm) were sampled at each of the six mangrove stands at Al Lith, South Jeddah, Dahaban, Thuwal, Rabigh and Mastorah. Sampling was conducted once per month from May 2019 to April 2020. Leave samples were collected from 15 mangrove trees at each site for analyses of nutrients, stable isotopes and antioxidants, while 15 surface sediment samples were collected for analyses of nutrient, stable isotopes, and sediment grain sizes. The samples were stored in clean zip lock bags, placed inside an ice cooler box, and conveyed to the laboratory. The sediments were sampled using Van Veen grab-250 cm^2^. At the six mangrove stands, in*-situ* measurement of physicochemical parameters such as salinity, pH, surface seawater temperature, dissolved oxygen (DO) was performed using a handheld YSI 556 MPS multi-parameter meter. Surface temperature and salinity were used to determine seawater density [32].

### Analyses of phosphorus, total organic matter and nitrogen in leaves and sediments

Oven-dried (40°C for 48 h) matured leaves of *A*. *marina* were ground into powder using an agate mortar and pestle, then sieved the remains with a 53 μm sieve. We digested 0.2 g of the ground leaf in HNO_3_ and H_2_O_2_ (3:1) at 180°C for 45 min. We weighed 0.4 g of dried sediments and placed these samples into a 50 ml digestion vessel, then added 8 ml of HNO_3_: HCl (1:1). The vessel was placed inside Anton-Paar PE Multiwave 3000 microwave oven and digested at 200°C for about 1 hr [33]. The vessel was filled by adding Ultrapure Millipore Q water and placed on a shaker for 24 h. A GF/F filter (Whatman) was used to filter the solution, and later total phosphorus (TP) concentration was analyzed in the filtrate using a Varian 720-ES (ICP-OES) inductively coupled plasma-optical emission spectrometer.

A FLASH 2000 CHNS analyzer was used to determine total organic carbon (TOC) and total nitrogen (TN) after 2 mg of ground leaves were weighed and loaded into a tin capsule [34]. The Semimicro-Kjeldahl and K_2_Cr_2_O_7_/H_2_SO_4_ oxidation methodological protocol were followed to analyze nitrogen and carbon contents in sediments [35].

### Grain size analyses

The total dry weight of oven-dried sediment samples was determined. First, distilled water was used to soak the dried sediments overnight to disintegrate the solidified aggregates. Wet sediments were washed gradually and passed through 0.063-mm and 2-mm sieves to separate fractions of gravel (>2 mm), coarse grain (0.063–2 mm) and mud (clay and silt, <0.063 mm). The fractions of the residue obtained in the sieves were weighed after drying at 40°C to determine different sediment grain size percentages [36].

### Analyses of stable carbon and nitrogen isotope

The protocol described in Gong and Zhang [37] was used to measure stable isotopes. Carbonate was removed from the dried sediment samples by treatment with 10% HCl (v/v) and rinsed with ultrapure Millipore Q water until a pH of 7.0 was reached. The sediment samples were then ground after drying in an oven at 50ºC. Samples of dry leave were ground and homogenized and then directly analyzed. Bulk organic matter δ^13^C and δ^15^N were determined using a Thermo elemental analyzer–ConFlo IV–Delta V Advantage mass spectrometer in Ocean College, Zhejiang University. All results for δ^13^C and δ^15^N were reported to V-PDB and air-N_2_, respectively. The expected standard deviation was less than 0.2‰.

### Measurement of CAT, GST and SOD in *A*. *marina*

Distilled water was used to wash the *A*. *marina* leaves to remove debris, and the leaves were pulverized in an ice-cold mortar and pestle with a 0.01(M) phosphate buffer (pH 7), then centrifuged at 14,000 rpm 4°C for 24 min [27, 38]. After centrifuging, the supernatant was used to measure antioxidant enzyme activities with a Labtronics spectrophotometer (Model: LT-291 Single Beam UV-VIS).

CAT activity was measured using the reaction mixtures containing 0.01 M phosphate buffer, 30 mM hydrogen peroxide, and the enzyme extract. It was measured at an absorbance of 230 nm for 2 min., in *μ*mol/min/mg protein.

The reaction mixture used for assay of GST activity was made of 1 mM 1-chloro 2,4-dinitrobenzene (CDNB), 0.1 M potassium phosphate buffer at pH 6.5, 1 mM-40 mM GSH, and 100 μL of leaf extracts in a total volume of 1 mL. Activities were measured at 340 nm per min, after the production of GS-DNB, and were recorded with a spectrophotometer at 25°C [39].

The protocol described by Keyster et al. [40] was used for the assay of SOD. We added 10 μl of the enzyme extract to a reaction mixture containing 0.1 mM xanthine, 6.25 nM xanthine oxidase, 50 mM K_2_HPO_4_, pH 7.8, 0.1 mM EDTA, 0.025% (w/v) Triton X-100, and 0.1 mM 2-(4-iodophenyl)-3-(4-nitrophenyl)-5-(2,4-disulfophenyl)-2H-tetrazolium (WST-1). SOD activity was measured on a spectrophotometer at an absorption of 450 nm at 37°C for 20 min. The specific activity was recorded as units/mg protein, where 1U of enzyme activity is the enzyme concentration required to avoid 50% production of chromogen under conditions of the assay for 1 minute.

### Data analysis

The hypotheses tested in this study are (i) if nutrients in sediment and mangrove *A*. *marina* sediment grain sizes, physicochemical parameters and antioxidant enzyme activities varies significantly across the sites and (ii) if a significant relationship exists between nutrients in sediments and *A*. *marina*, sediment grain sizes, physicochemical parameters and antioxidant enzyme activities. The predictions were based on possible salinity and nutrient stress at Rabigh site as it receives land runoff from agricultural fields about 5 km away from its southern end, livestock activities and decreased seawater circulation at its northern end. In addition, stress due to nutrient limitation and anthropogenic activities such as extraction of resources at the mangroves and agricultural activities approximately 7 km away from Al lith, South Jeddah, Dahaban and Thuwal sites were predicted as causal of stress. In contrast, the Mastorah site was 50 km away from huge industrial activities and was the only possible cause of stress predicted.

Data analysis was performed after initial homogeneity of variance and a test of normality was carried out using Levene’s homogeneity of variance and Shapiro-Wilk tests, respectively. One-way analysis of variance (ANOVA; α = 0.05) was used to determine significant differences in nutrients in sediments and *A*. *marina*, sediment grain sizes, physicochemical parameters and antioxidant enzyme activities across the six mangrove stands. Where statistical significance occurred, Tukey’s post-hoc test was used for mean separation at *P* < 0.05. Correlation-base dimensional principal component analysis (PCA) using the factoextra package and Pearson correlation were employed to test the relationship between nutrients in sediments and *A*. *marina*, sediment grain sizes, physicochemical parameters and antioxidant enzyme activities. Before the PCA analysis, the index was set at correlation, and relationships between variables were deduced from the PCA pattern after analysis. Data analysis was achieved using R for Windows (v. 4.0.3).

## Results and discussion

### Changes in environmental parameters and percentage of mud and sand in study sites

Physicochemical parameters such as salinity, temperature, DO, pH and density vary across the six different sites in this study selected at the central Red Sea (Table 2). Seawater surface temperatures range from 26.89°C at Thuwal to 29.32°C at Rabigh. Salinities in the six sites vary from the lowest value of 38.81 at Thuwal to the highest value of 40.75 at Rabigh. The seawater surface temperature and salinity results with high S.E. revealed frequent changes in these parameters in the sites across time. DO measurements ranged from 5.21 mg/L at Rabigh to 6.41 mg/L at Thuwal. However, there exists no significant difference in DO values recorded at Al lith, South Jeddah, Dahaban, Thuwal and Mastorah. Minimal variation was established for pH values, with the lowest value of 8.11 at Thuwal and the highest value of 8.49 at Rabigh. Seawater surface density range from 1025.59 kg m^-3^ at South Jeddah to 1026.28 at Rabigh (Table 2).

**Table 2:** Mean physicochemical properties of seawater and mangrove heights at 6 sites on the coast of the central Red Sea, Saudi Arabia.

			Study Sites			
	Al Lith	South Jeddah	Dahaban	Thuwal	Rabigh	Mastorah
**Temperature (°C)**	27.16±2.83c	27.92±4.24b	26.92±4.21cd	26.89±3.54d	29.32±3.95a	27.01±3.01cd
**Salinity (‰)**	39.47±4.98b	39.21±3.43bc	38.95±3.21c	38.81±5.14	40.75±5.20a	39.02±2.98c
**D (kg m** ^**-3**^ **)**	1026.04±141.40b	1025.59±282.80d	1025.73±324.30c	1025.63±324.30cd	1026.28±282.80a	1025.75±253.60c
**DO (mg/L)**	6.03±0.21c	6.05±0.46c	6.33±0.52b	6.41±0.62a	5.21±0.81d	6.00±0.72c
**pH**	8.34±1.05a	8.32±0.99a	8.23±1.12a	8.11±1.12a	8.49±1.91a	8.30±1.55a
**MH (m)**	2.01±0.01d	2.05±0.01d	2.45±0.23c	3.21±0.22a	3.14±0.32b	3.15±0.42b

DO—Dissolve oxygen, MH—Mangrove height, D—Density, BG—Between groups, WG—Within groups, TO—Total. a, b, bc, c, cd, d = Mean rankings, Mean ± S.E with different letters (a-d) along the same row were significantly different (*P* < 0.05).

Significant differences (*P* < 0.05) in the percentages of different grain sizes of sediment classes were recorded among study sites (Table 3). The coarse grain (0.063–2 mm) percentage in sediments range from 45.50% at Rabigh to 88.83% at South Jeddah, clay silt particles (< 0.063 mm) ranged from 10.53% at South Jeddah to 54.29% at Rabigh, while gravels (> 2mm) range from 0.20% at Rabigh and Dahaban to 0.63% at South Jeddah (Table 4). These results were used to classify sediment type (see Table 4).

**Table 3:** F values and corresponding significance level (p values) after ANOVA test for physicochemical parameters, nutrients and antioxidants.

Physicochemical Property							
	Temperature (°C)	Salinity (‰)	D (kg m^-3^)	DO (mg/L)	pH	MH (m)	
F value	451.27	183.95	126.11	267.42	1.42	3222.57	
P value	0.00	0.00	0.00	0.00	0.33	0.00	
Source of variation	Between groups		Within groups		Total		
df	5		6		11		
**Nutrient in Sediment**							
	**C**	**N**	**P**	**C:N**	**N:P**	**δ** ^**13**^ **C**	**δ** ^**15**^ **N**
F value	3236.09	1003.61	1003.61	132.18	20.29	1364.29	22.81
P value	0.00	0.00	0.00	0.00	0.01	0.00	0.00
Source of variation	Between groups		Within groups		Total		
df	5		84		89		
**Grain size**							
	**CS (0.063–2 mm)**	**CSTP (< 0.063mm)**	**G (> 2mm)**				
F value	113.59	114.82	6.30				
P value	0.00	0.00	0.00				
Source of variation	Between groups		Within groups		Total		
df	5		12		17		
**Nutrient in Leaves**							
	**C**	**N**	**P**	**C:N**	**N:P**	**δ** ^**13**^ **C**	**δ** ^**15**^ **N**
F value	8.55	10.62	10.62	7.54	6.78	9.78	25.62
P value	0.00	0.00	0.00	0.00	0.00	0.00	0.00
Source of variation	Between groups		Within groups		Total		
df	5		84		89		
**Antioxidants**							
	**CAT**	**GST**	**SOD**				
F value	65.25	387.61	5.92				
P value	0.00	0.00	0.01				
Source of variation	Between groups		Within groups		Total		
df	5		6		11		

**Table 4:** Mean (± SE) of nutrients concentrations (%) in mangrove sediment and grain size at 6 sites on the coast of the central Red Sea, Saudi Arabia.

(n = 90)	Al Lith	South Jeddah	Dahaban	Thuwal	Rabigh	Mastorah	Average
**C**	0.36±0.04d	0.32±0.02de	1.02±0.07c	1.80±0.03b	1.92±0.04a	0.08±0.005f	0.92±0.13
**N**	0.03±0.001d	0.02±0.004de	0.22±0.003c	0.24±0.007b	0.38±0.008a	0.01±0.004e	0.15±0.06
**P**	0.013±0.001d	0.012±0.0002de	0.083±0.003c	0.085±0.007b	0.145±0.03a	0.003±0.0003e	0.06±0.01
**C:N**	11.35±1.66b	15.41±1.37a	4.74±0.08d	7.71±0.26c	5.14±0.12d	7.60±0.30c	8.66±1.65
**N:P**	2.31±0.51bd	1.69±0.33e	2.65±0.41c	2.82±0.36b	2.62±0.18c	3.33±0.62a	2.57±0.23
**δ** ^**13**^ **C**	-23.24±0.08d	-18.87±0.06c	-13.68±0.09a	-19.02±0.12c	-13.62±0.14a	-17.14±0.06b	-17.60±1.49
**δ** ^**15**^ **N**	2.47±0.62d	2.31±0.76d	2.81±0.39c	3.04±0.99b	4.39±0.51a	2.03±0.83e	2.84±0.49
**CS (0.063–2 mm)**	83.53±12.54a	88.83±13.48a	65.37±13.95c	52.53±9.06d	45.50±7.52e	72.48±11.66b	68.04±6.95
**CSTP (< 0.063mm)**	15.97±2.55e	10.53±1.32e	34.42±2.17c	47.15±6.82b	54.29±9.31a	27.06±5.16d	31.57±7.01
**G (> 2mm)**	0.49±0.05ab	0.63±0.06a	0.20±0.08c	0.31±0.06bc	0.20±0.01c	0.45±0.03ab	0.38±0.07
**Texture**	Sand	Sand	Sand	Sand	Sandy Loam	Sand	

C—Total organic carbon, N—Total nitrogen, P—Total phosphorus, C:N–Carbon-Nitrogen ratio, N:P–Nitrogen -Phosphorus ratio, CS—Coarse sandy, SSCS—Clay and Silt particles, BG—Between groups, WG—Within groups, TO–Total. a, b, bc, c, cd, d, de, e, f = Mean rankings. Mean ± S.E with different letters (a-d) along the same row were significantly different (*P* < 0.05).

The amount of rainfall in different coastal regions has considerable implications on changes in such environmental parameters as salinity, temperature and pH. This observation applies to the entire central Red Sea region with the dry season and annual average rainfall of only about 0.5 mm and 51.2 mm respectively [41]. This could give an insight into the possible frequently changing seawater surface temperature and salinity in mangrove sites across time [41]. High solar radiation is the primary reason for high temperature in the central Red Sea. With intense evaporation and decreased rainfall, the resultant decrease in land runoff in most study areas exacerbated high salinity levels [42]. However, the inverse trend of DO with temperature and salinity in this study results from the effect of salinity and temperature on oxygen dissolution [43]. The Rabigh mangrove ecosystem was unique among the six study areas with other variables indicative of its pristine nature. Restricted circulation with the tendency of even greater evaporation contributed to high salinity and temperature, with a resultant increase in seawater surface density (1026.28 kg m^-3^) relative to other mangrove ecosystems.

The lowest DO at Rabigh results from decreased solubility in the warmer water body [42], resulting in a predominance of clay and silt sediment texture and high nutrients in the sediments.

### Factors for nutrients distribution in mangrove stands

The concentrations of TOC, TN and TP were vastly higher in mangrove leaves than those in sediments (Table 5). However, TOC, TN and TP in sediments at Rabigh were significantly (*P* < 0.05; Table 3) higher compared to the concentrations recorded at Al lith, South Jeddah, Dahaban, Thuwal and Mastorah mangrove stands (Table 4).

**Table 5:** Mean (± SE) of nutrients concentrations (%) and antioxidants (*μ*mol/min/mg protein) in *A*. *marina* leaves at 6 sites on the coast of the central Red Sea, Saudi Arabia.

(n = 90)	Al Lith	South Jeddah	Dahaban	Thuwal	Rabigh	Mastorah	Average
**C**	42.57±4.98d	43.26±3.92cd	42.09±3.09d	44.95±5.84ab	43.94±5.37bc	45.34±3.41a	43.69±0.53
**N**	1.20±0.05cd	1.15±0.03d	1.36±0.02b	1.28±0.04bc	1.52±0.03a	1.28±0.05bc	1.30±0.05
**P**	0.64±0.03cd	0.55±0.02d	0.77±0.01b	0.71±0.02bc	0.84±0.04a	0.69±0.03bc	0.70±0.04
**C:N**	36.64±3.69a	37.77±4.12a	31.09±3.55b	35.46±4.27a	29.11±3.70b	36.29±2.41a	34.40±1.41
**N:P**	1.86±0.21b	2.09±0.48a	1.77±0.32d	1.80±0.16c	1.81±0.22c	1.88±0.21b	1.87±0.05
**δ** ^**13**^ **C**	-27.92±0.13b	-27.62±0.10b	-27.08±0.16b	-27.03±0.18a	-26.94±0.17a	-27.08±0.11a	-27.41±0.18
**δ** ^**15**^ **N**	2.02±0.26d	2.00±0.54d	2.21±0.17c	2.57±0.26a	3.09±0.19a	1.97±0.28e	2.31±0.42
**CAT**	7.60±0.78b	7.45±0.24b	5.05±0.25d	4.98±±0.15d	9.15±0.92a	7.70±0.10c	7.08±0.62
**GST**	29.10±2.20b	28.77±2.96b	9.80±1.13d	9.13±1.10d	51.60±4.74a	23.95±2.96c	27.06±7.81
**SOD**	5.70±0.40c	6.35±0.51b	4.35±0.45d	3.56±0.46d	7.05±0.33a	5.55±0.51c	5.43±0.52

C—Total organic carbon, N—Total nitrogen, P—Total phosphorus, C:N–Carbon-Nitrogen ratio, N:P–Nitrogen-Phosphorus ratio, CAT–Catalase, GST—Glutathione–S- transferase, SOD—Superoxide dismutase, BG—Between groups, WG—Within groups, TO–Total. a, b, bc, c, cd, d, de, e = Mean rankings. Mean ± S.E with different letters (a-d) along the same row were significantly different (*P* < 0.05).

Increasing concentrations of TOC, TN and TP in sediment among study areas were in the order Rabigh>Thuwal>Dahaban>Al lith>South Jeddah>Mastorah (ANOVA; *P* < 0.05; Table 3). TN in mangrove leaves at Rabigh was the highest and was about 1.3 times higher than at South Jeddah and far greater than in the sediments at Rabigh and South Jeddah (Table 4). The TP concentration in leaves at Rabigh was about 1.5 times higher than at South Jeddah and substantially lower than in the sediments (Table 4), signifying a lower enrichment factor of TP in leaves than the sediment.

**Fig 2:**
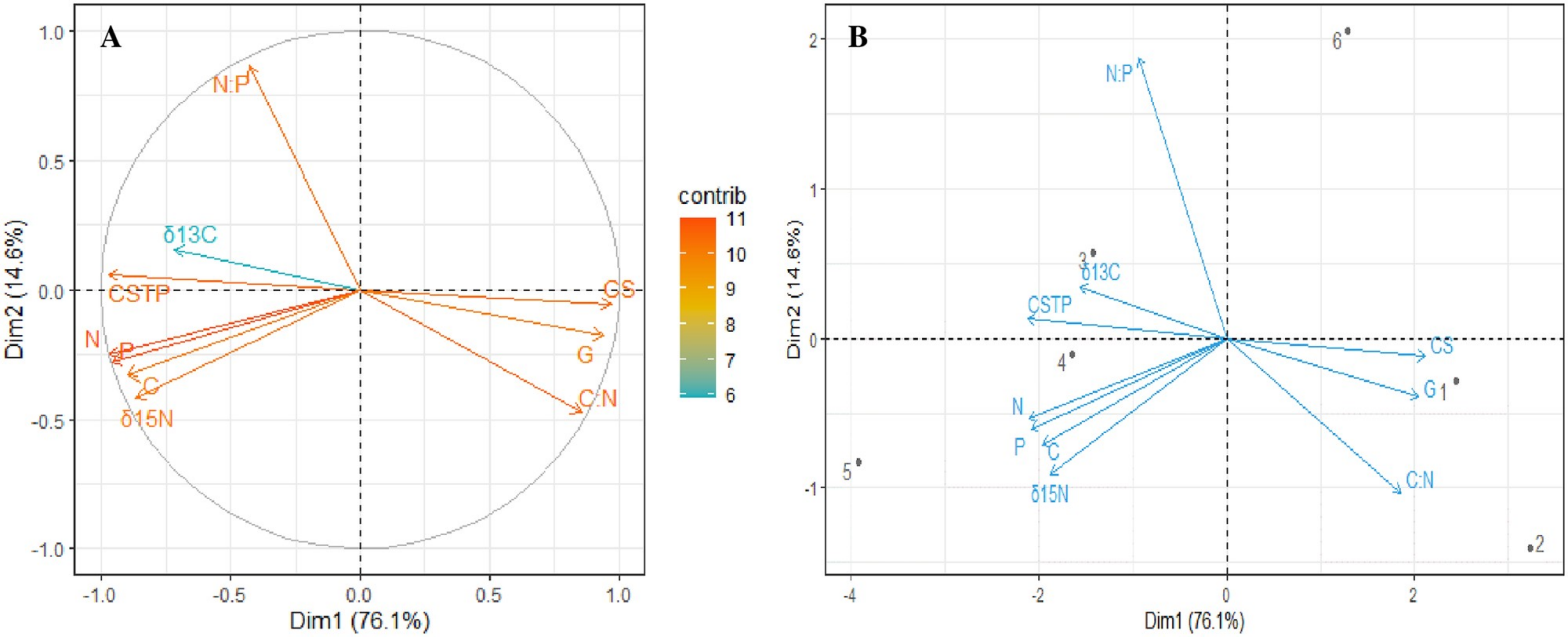
The (A) contribution plot and (B) biplot of a principal component analysis conducted to examine the relationship between sediment grain size and nutrients. C—Total organic carbon, N—Total nitrogen, P—Total phosphorus, δ^13^C—Carbon isotope, δ^15^N—Nitrogen isotope, C:N—Carbon-Nitrogen ratio, N:P—Nitrogen-Phosphorus ratio, CS—Coarse sandy, CSTP—Clay and Silt particles, G–Gravels, 1–8—Study areas (1-Al Lith, 2-South Jeddah, 3-Dahaban, 4-Thuwal, 5-Rabigh and 6-Mastorah).

The principal component analysis results for sediment grain size, C/N ratio, N/P ratio, stable isotope and nutrients indicate that dimensions 1 (76.1%) and 2 (14.6%) explain most of the variability (Fig 2A and 2B). Grouping of study areas is presented in Fig 2B, revealing Rabigh and Mastorah not forming a group with other study areas. There were similarities between Dahaban and Thuwal, and South Jeddah and Al lith.

The percentages of clay and silt are strongly associated with N/P, nutrients, and δ^13^C in the sediments, whereas coarse fractions and gravels in sediments are associated with C/N (Fig 2A and 2B). The type of sediment grain size varies substantially across the study areas. The highest percentages (88.83 and 0.63%) of coarse and gravel grain sizes at South Jeddah are not significantly different from those at Al lith. However, the lowest coarse grain size percentage was recorded at Rabigh with about 5 times more clay and silt grain sizes than at South Jeddah, suggesting that the sediment texture type at Rabigh be classified as loamy sand. Although, the sediment at Rabigh was about 45.50% and 0.20% of coarse sediment and gravel respectively, and the clay silt particles were only about 1.19 times of coarse sediment particles (Table 4). South Jeddah has the highest C/N ratio (15.41) and lowest N/P ratio (1.69) in sediments, and the highest C/N (37.77) and N/P ratio (2.09) in mangrove leaves. However, Dahaban has the lowest C/N ratio (4.74) in sediment, which was not substantially different from C/N ratio (5.14) at Rabigh. The lowest C/N ratio and N/P ratio in mangrove leaves were recorded at Rabigh and Dahaban, respectively and were about 1.30 and 1.18 times that of South Jeddah (Table 5).

**Fig 3:**
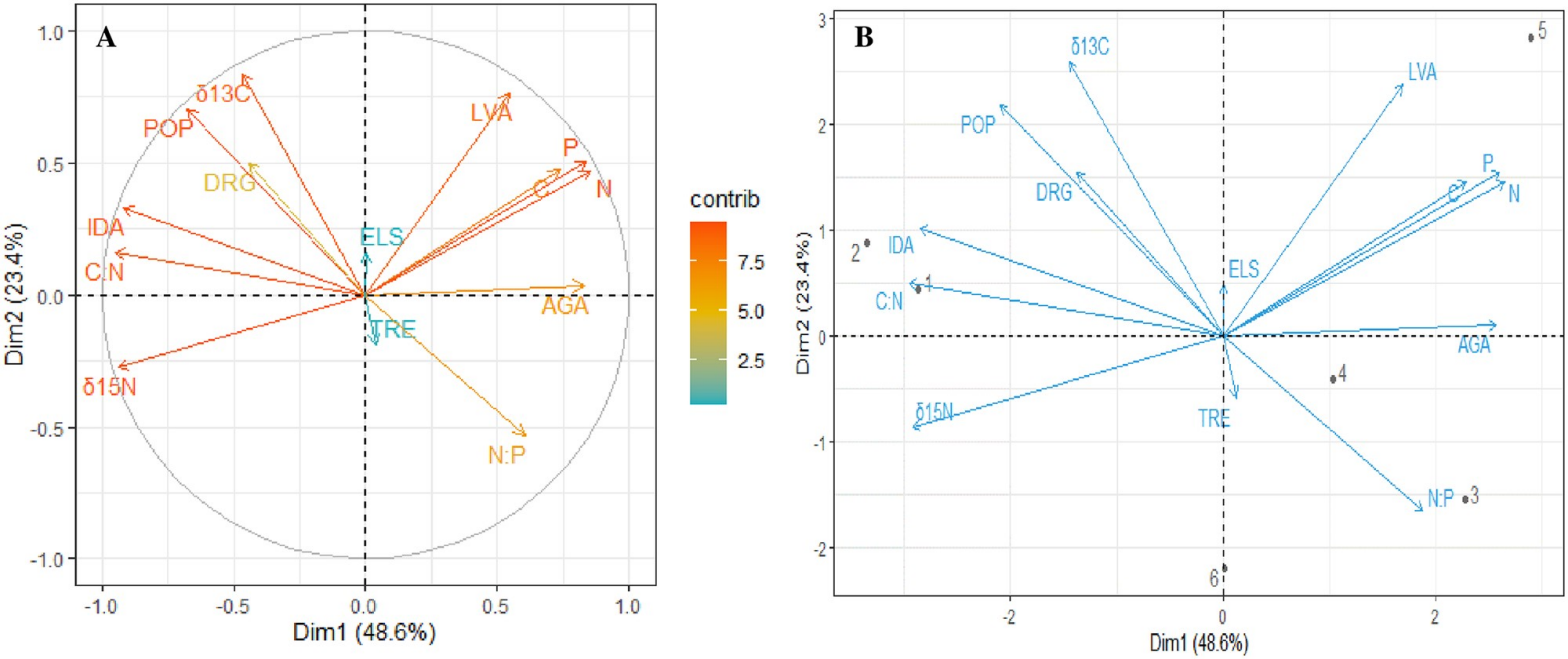
The (A) contribution plot and (B) biplot of a principal component analysis conducted to examine the relationship between nutrients and anthropogenic activities. C—Total organic carbon, N—Total nitrogen, P—Total phosphorus, δ^13^C—Carbon isotope, δ^15^N—Nitrogen isotope, C:N—Carbon-Nitrogen ratio, N:P—Nitrogen-Phosphorus ratio, POP–Human population, DRG—Dredging, ELS—Extraction of living resources (Fishing), TRE- Tourism and recreation, LVA—Livestock activities, AGA—Agricultural activities, IDA—Industrialization, 1–8—Study areas (1-Al Lith, 2-South Jeddah, 3-Dahaban, 4-Thuwal, 5-Rabigh and 6-Mastorah).

In each study site, we classified types of land use and predominant anthropogenic activities. These factors vary from one mangrove ecosystem to another. However, PCA components 1 and 2 explained 73% of the total variation, suggesting that anthropogenic activities influence nutrients (Fig 3A and 3B). Factors such as human population, industrialization, agricultural activities, livestock activities had a higher contribution (>7) to the total variation (Fig 3A).

The substantial differences in organic carbon, nitrogen and phosphorus in the sediments across the study areas are most likely due to anthropogenic impact, including socio-economic and environmental transformations, urban effluent, land runoff containing fertilizers, extraction of living resources such as fishing, among others [4, 5]. Restricted circulation, sediment texture, the input of land runoff containing agricultural fertilizer and camel grazing are the key reasons for higher N and P enrichment in Rabigh site than the others.

Our results showed that TN and TOC in sediment at Rabigh were 38 and 24 times the concentrations at Mastorah. This suggests a higher increase in TN than TOC, which can be explained by stimulation of soil C remineralization by urban effluents or land runoff as reported elsewhere in a tropical mangrove ecosystem [44]. Further, phosphate at Rabigh was 48 times the value at Mastorah, which had the lower concentration of phosphate among the mangroves. It is worth noting that the average TN and TP for the six mangroves were 2.5 and 6 folds the values (TN = 0.06% and TP = 0.01%) reported in a conserved subtropical mangrove forest in southeastern Australia dominated with *A*. *marina* [3]. This suggests the influence of anthropogenic activities such as agricultural activities in the catchment of the six mangroves under study [4]. Congruent to Almahasheer et al. [13], TN and TP in leaves were higher than the concentrations in sediment; however, the average TN in leaves at our study sites was about 50% of that (1.84%) reported earlier in some oligotrophic areas of the central Red Sea. TP (0.066%) in oligotrophic areas of the central Red Sea was 10% of that reported in our study [45], suggesting more phosphorus enrichment than nitrogen.

Higher clay and silt particles in Rabigh and Thuwal relative to the other mangrove stands coincide with their high nutrient concentrations. Clay particles have been reported to have a high nutrient absorption capacity in the form of organic molecules [46–48]. In addition, the water residence time in these two mangrove stands, especially Rabigh, when compared to other mangroves, might have allowed the dissolved and particulate nutrients in surface water settled to the benthic region [36, 49]. This is unlike other study sites because of its rapid circulation and low water residence time that may decrease the settling of the nutrients to the benthic regions of seawater in the mangrove ecosystems. The unique case of the Rabigh mangrove stand is the restricted inflow of seawater that resulted in the stagnation of water and low DO reported in this study. The input of land runoff from Rabigh City and Wadi Rabigh due to seasonal flood events [50], containing agricultural waste and restricted circulation, has given more insight into the increase in nutrients in this ecosystem than the others. Additionally, fecal pellets from livestock such as camel during grazing on mangrove plants tend to add to N and P in sediments, as reported in southern Australia [51].

### Environmental factors for stress in *A*. *marina*

**Fig 4:**
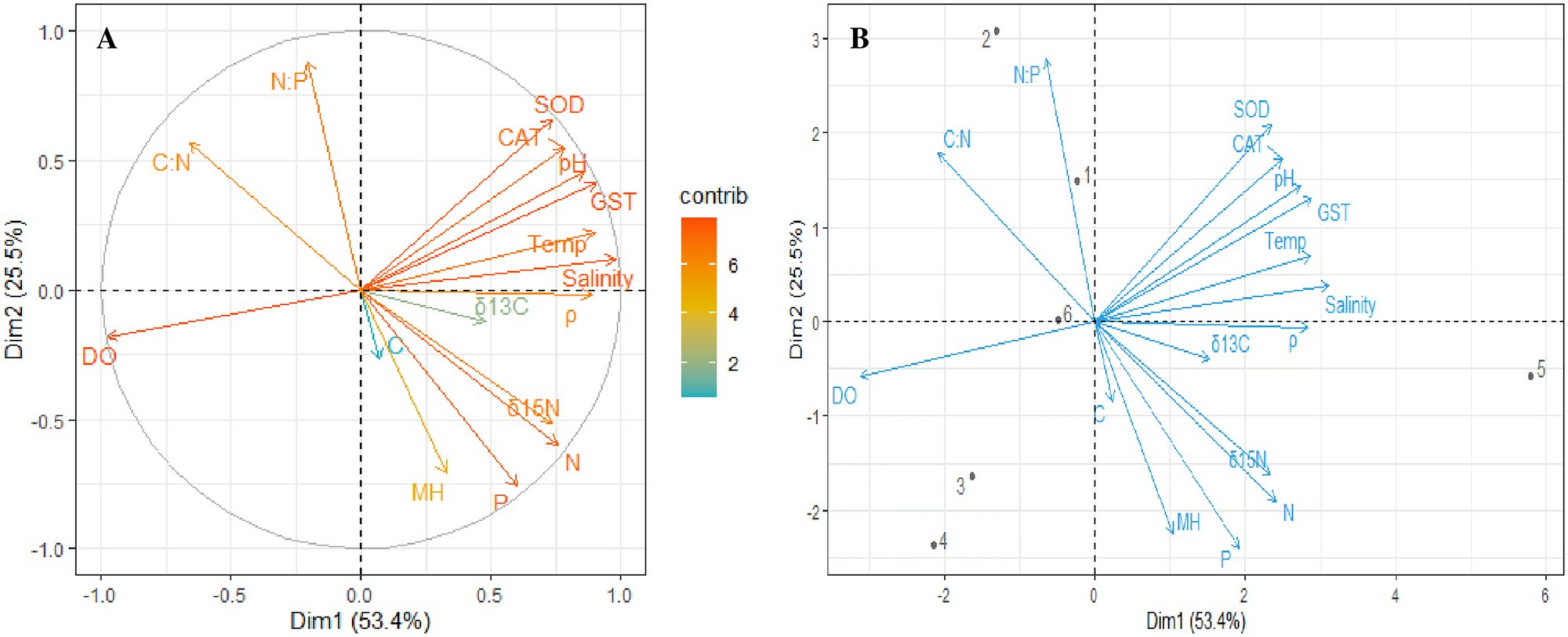
The (A) contribution plot and (B) biplot of principal component analysis in which the relationships among nutrients, physicochemical parameters, antioxidants and mangrove height on the coast of the central Red Sea, Saudi Arabia, were examined. C—Total organic carbon, N—Total nitrogen, P—Total phosphorus, δ^13^C—Carbon isotope, δ^15^N—Nitrogen isotope, C:N—Carbon-Nitrogen ratio, N:P—Nitrogen-Phosphorus ratio, CAT—Catalase, GST—Glutathione S- transferase, SOD—Superoxide dismutase, DO—Dissolve oxygen, MH—Mangrove height, Temp—Temperature, *ρ–*Density, 1–8—Study areas (1-Al Lith, 2-South Jeddah, 3-Dahaban, 4-Thuwal, 5-Rabigh and 6-Mastorah).

The antioxidant enzymes CAT (9.15 *μ*mol/mg protein), GST (51.60 *μ*mol/mg protein) and SOD (7.05 *μ*mol/mg protein) activities served as biomarkers of oxidative stress among all the study areas were highest in mangroves at Rabigh (Table 3). However, mangroves at Thuwal had the lowest CAT (4.98 *μ*mol/mg protein), GST (9.13 *μ*mol/mg protein) and SOD (3.56 *μ*mol/mg protein), but was not significantly different from the antioxidants activities in mangroves at Dahban. Correlation-based PCA revealed a strong positive correlation between TOC (r = 0.8549), TN (r = 0.8934) and TP (r = 0.8650) in leaves of *A*. *marina* and antioxidant enzymes activity (Fig 4A and 4B). A strong negative relationship was established between dissolved oxygen (r = -0.9688) and antioxidants (CAT, GST and SOD), salinity, and temperature, while a weak positive relationship was established between mangrove height (r = 0.3719) and antioxidants (CAT, GST and SOD), salinity, and temperature (Fig 4A and 4B). Dimensions 1 and 2 contributed 53.4% and 25.5% of the total variation (Fig 4A and 4B).

Pearson correlation revealed a significant positive correlation between TOC, TN, and TP with GST, SOD and CAT respectively. A strong negative correlation was revealed between DO and CAT, GST, SOD, temperature and salinity. Further, a strong positive correlation was revealed between salinity and CAT, GST and SOD (Table 6).

**Table 6:** Pearson correlation coefficient between potential stressors, antioxidants and mangrove height at 6 sites on the coast of the central Red Sea, Saudi Arabia.

	**C**	**N**	**P**	**C:N**	**N:P**	**δ** ^**13**^ **C**	**δ** ^**15**^ **N**	**CAT**	**GST**	**SOD**	**Temp**	**Salinity**	**ρ**	**DO**	**pH**	**MH**
**C**	1															
**N**	0.092	1														
**P**	0.078	0.965**	1													
**CN**	0.200	-0.951**	-0.928**	1												
**NP**	-0.035	-0.662	-0.832*	0.647	1											
**δ13C**	0.888*	0.470	0.400	-0.198	-0.183	1										
**δ15N**	-0.187	-0.927**	-0.911*	0.891*	0.634	-0.535	1									
**CAT**	0.776	0.739	0.863*	-0.122	0.341	0.272	0.029	1								
**GST**	0.854*	0.522	0.322	-0.422	0.097	0.375	-0.289	0.935**	1							
**SOD**	0.387	0.893*	0.335	-0.136	0.453	0.115	0.059	0.970**	0.896*	1						
**Temp**	0.001	0.577	0.355	-0.522	0.141	0.394	-0.450	0.804	0.936**	0.810	1					
**Salinity**	-0.067	0.666	0.499	-0.604	-0.112	0.331	-0.445	0.821*	0.966**	0.880*	0.930**	1				
**ρ**	-0.140	0.648	0.578	-0.591	-0.389	0.189	-0.356	0.691	0.823*	0.603	0.676	0.899*	1			
**DO**	-0.096	-0.636	-0.448	0.525	0.013	-0.439	0.407	-0.897*	-0.987**	-0.852*	-0.926**	-0.967**	-0.837*	1		
**pH**	-0.164	0.419	0.227	-0.375	0.170	0.109	-0.123	0.942**	0.939**	0.952**	0.830*	0.888*	0.795	-0.925**	1	
**MH**	0.804	0.619	0.643	-0.392	-0.527	0.896*	-0.704	-0.033	0.128	-0.184	0.147	0.158	0.137	-0.246	-0.093	1

*. Correlation is significant at the 0.05 level (2-tailed),

**. Correlation is significant at the 0.01 level (2-tailed), ρ–Density, C—Total organic carbon, N—Total nitrogen, P—Total phosphorus, C:N–Carbon-Nitrogen ratio, N:P–Nitrogen-Phosphorus ratio, CAT–Catalase, GST—Glutathione–S- transferase, SOD—Superoxide dismutase, Temp–Temperature, DO–Dissolved oxygen, MH–Mangrove height.

Therefore, salinity, then temperature and nutrients are the most important contributing factors to the total variation in the Rabigh mangrove ecosystem. These characteristics are negatively correlated with DO and positively correlated with mangrove height (Fig 4A and 4B).

The cellular damage and photosynthesis inhibition are caused by the reactive oxygen species (ROS) such as H_2_O_2_, OH and O_2_ produced during oxidative stress, which is triggered by abiotic stress factors such as high salinity, high temperature, and nutrient limitation [25, 52]. Such antioxidant enzymes as superoxide dismutase catalase (CAT), Glutathione S-transferase (GST), superoxide dismutase (SOD) and peroxidase (POD) are some of the antioxidative defense system used by plants, including mangroves, to scavenge excess ROS produced in a stressed condition. This system terminates the chain of lipid peroxidation [53]. Thus, antioxidants serve as early warning signals of physiological response to stress conditions in mangroves [26, 27, 54, 55].

There were significant differences in CAT, GST and SOD measurements within mangrove leaves at different study sites, with the highest mean antioxidant activities at Rabigh, South Jeddah and Al lith. However, positive correlations between TOC, TN and TP and the three antioxidants suggest that stress in these mangrove stands might be attributable to nutrient limitation [56]. Sanders et al. [8] reported a high TN (9.25%) and TP (2.13%) in a conserved mangrove forest with *Avicennia* sp. in southeastern Brazil. Elsewhere, *A*. *marina* stands with the highest stem density, biomass and height (12.9 m) in Matang Mangrove Forest Reserve in Malaysia were reported to have a very high TN (12.01%) and TP (0.276%) [57]. In contrast, Dangremond and Felle [58] found out lower values of 0.02% and 0.006% for TN and TP in the Caribbean population of the rare mangrove *Pelliciera rhizophorae*. Additionally, TN and TP in the stunted mangrove trees of *A*. *marina* leave in the oligotrophic central Red Sea were only 0.84–2.89%, and 0.007–0.12%, respectively, while 0.025–0.639% and 0.014–0.098% for TN and TP, respectively in sediments [45].

It is important that the average value of TN (1.3%) in leaves among the six mangrove stands was about 11% of that in healthy mangrove leaves in Malaysia [57] and 14% of that in a conserved mangrove forest in southeastern Brazil [8]. On the other hand, TP values were 25% and 33% of that in Malaysia [57] and southeastern Brazil [8]. This suggests that N limitation was one of the key causes for stress in the six mangrove stands. However, the mangrove stand at Rabigh is in a more favorable condition in terms of nutrient content in comparison to the other five ecosystems and those reported by Anton et al. [45] from the oligotrophic sites on the coast of the central Red Sea.

The Rabigh site is different from the other five mangrove sites, even in terms of water residence time (Table 1), physicochemical parameters (high salinity, high temperature, low DO and high seawater density), anthropogenic impact, N and P in leaves and sediments. TN (0.38%) and TP (0.145) (Table 4) in sediments were the highest among the six study areas. However, N in leaves was about 1/6 and 1/8 of that in conserved mangroves in southeastern Brazil and healthy mangroves in Malaysia. P content in leaves was even greater than the latter, signifying N limitation may not be a fundamental contributor for the major stress in mangroves at the Rabigh mangrove ecosystem. The lowest DO observed at Rabigh mangrove stand relative to the other five mangrove stands (Table 2) was presumably due to the presence of stagnant water and bacterial community metabolism [59]. However, it also coincides with antioxidants activities and weak positive correlation (r = 0.3276) with mangrove height (Fig 4A and 4B), suggesting the effect of low DO could impact the whole mangrove ecosystem complexes and exacerbate stress in mangroves [49, 60].

The weak positive correlation between mangrove height and both salinity and temperature was presumably due to higher concentrations of salinity at these sites, which might have contributed to reduced nitrogen uptake efficiency and potassium uptake inhibition. This may have caused lipid peroxidation and oxidative damage or stress in *A*. *marina* [55, 61]. Higher nutrient concentrations were reported to enhance the salt tolerance of mangroves and contributed to increased mangrove height despite high salinity [62]. However, other factors such as heavy metals reported in Red Sea mangroves [13] can synergize with salinity to contribute to oxidative stress in such ecosystems [55]. High temperatures in this ecosystem constitute a type of abiotic stress that can affect the developmental, physiological, and biochemical integrity of mangroves [63]. Exposure of mangroves to elevated temperatures (i.e., >25°C) has triggered abscission of leaves, growth inhibition of shoot and fruit damage [64]. This most likely could be the reason for a positive correlation (r = 0.9012) between temperature and the antioxidants (CAT, GST and SOD) activities in *A*. *marina* at our sites.

### Stable isotopes of carbon and nitrogen in sediments and mangrove leaves

**Fig 5:**
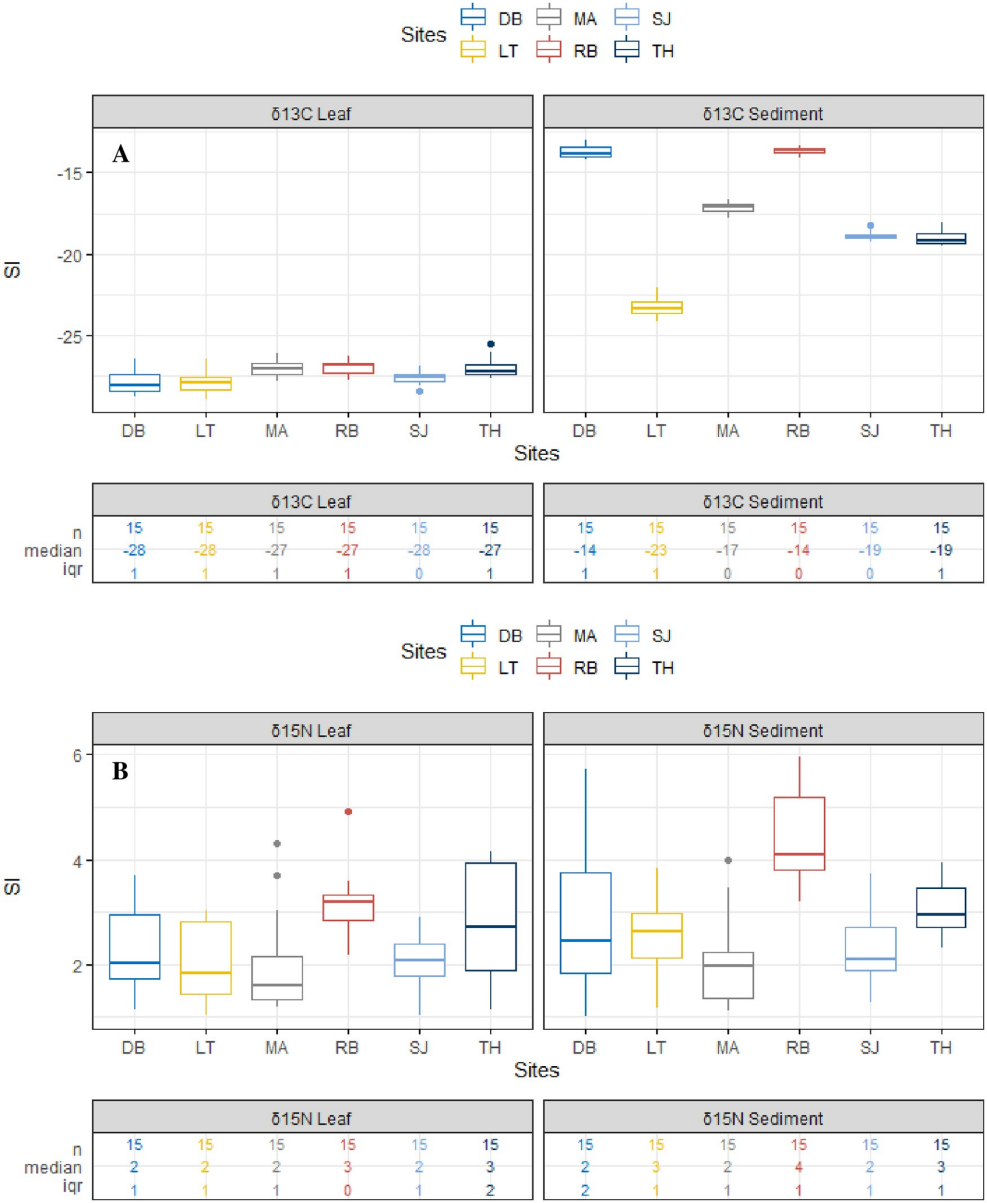
Boxplots showing (A) δ^13^C and (B) δ^15^N variability in sediment and *A*. *marina* leaves for the study sites on the coast of the central Red Sea and their corresponding median values. SI–Stable isotope value, iqr–interquartile range, LT-Al Lith, SJ-South Jeddah, DH-Dahaban, TH-Thuwal, RB-Rabigh and MA-Mastorah.

The stable isotopes of carbon δ^13^C in mangrove leaves ranged from -27.92‰ to -26.94‰, with a significant difference among our study sites (Table 5). δ^13^C values in leaves at Al lith were the lowest, but with no significant difference with the values at Dahaban and South Jeddah. The sediment’s δ^13^C values were substantially higher than those in mangrove leaves (Table 4, Fig 5A). Median values for δ^13^C across the study sites in sediment and leaves are presented in Fig 5A. The values of δ^15^N in mangrove leaves ranged from 1.97‰ at Mastorah to 3.09‰ at Rabigh (Table 5). For sediment, the lowest value of δ^15^N was 2.03 at Mastorah, and the highest was 4.39 at Rabigh and were 1.03 and 1.42 greater than the values in mangrove leaves. Thus, sediments had higher δ^15^N values of about ~1‰ than the mangrove leaves across the six sites (Fig 5B).

δ^13^C values in the mangrove sediments were positively correlated with organic carbon (r = 0.8372), total nitrogen (r = 0.9451), total phosphate (r = 0.941) and negatively correlated with C/N (-0.8543). However, δ^15^N in sediments correlated positively with N/P (r = 0.8644) (Fig 2A and 2B). The values of δ^13^C and δ^15^N were associated with mangrove height and dissolved oxygen (Fig 4A and 4B), especially at the Rabigh mangrove stand (Fig 4B). Moreover, a negative correlation existed between N/P (r = -0.2312) and δ^15^N (Fig 4A and 4B).

Heavier δ^13^C in sediment recorded at Rabigh was more significant than that at Al lith by ~10‰. The value of δ^13^C for Al lith is the lowest among the six mangrove sites. However, δ^13^C in *A*. *marina* leaves at Rabigh was only greater than that at Al lith by ~1‰ (Table 5). The average value of δ^13^C in sediment among the six mangrove stands was higher than in leaves by ~10‰ (Fig 5A, Tables 4 and 5). Stress triggered by high salinity (40.75 ‰) (Table 2) could be the reason for the different or additional enrichment of δ^13^C in *A*. *marina* leaves across the mangrove stands, especially at Rabigh. Elsewhere, an increase in δ^13^C signatures from -33.81 to -28.41 ‰ was triggered by an upsurge in salinity concentration from 13 to 24 [65]. Stress in plants, including mangroves, exhibits a phenomenon known as stomatal conductance, which is an indirect consequence of stress and can result in intercellular CO_2_ pressure depletion [66]. However, since ^12^C is favorably assimilated due to discrimination during decarboxylation and diffusion, ^13^C enrichment in the assimilated material is caused by reduced CO_2_ pressure [65, 66].

The average δ^15^N value for the six mangroves is higher than those reported in *Laguncularia racemosa* in Florida, USA [67] (1.85–2.05‰) and *A*. *marina* in Australia (1.6–2.2‰) [68], possibly due to increased N cycling associated directly with anthropogenic impacts [8]. Agricultural waste is a product of anthropogenic activities and could contribute to an increase in δ^15^N values [57, 65, 69]. A possible influence of product of anthropogenic activities in mangrove ecosystems such as agricultural waste have been reported in a mangrove ecosystem in Matang mangrove ecosystem in Malaysia, New Zealand (5.4–9.8‰) [30], and other impacted sites located along the coast of the Red Sea (~4‰) [18].

## Conclusions

The results presented in this research indicate significant variation in carbon and nutrients (TN and TP) among six mangrove ecosystems in the central Red Sea part of Saudi Arabia. The differences may be associated with environmental impacts such as agricultural waste, extraction of natural resources, hydrological events, and changes in physicochemical parameters such as salinity and temperature. There were higher TN and δ^15^N in Rabigh, presumably due to stagnant water, runoff bringing agriculture waste from catchments and widespread livestock activities such as camel grazing.

N limitation and possibly salinity are likely sources of stress in Al lith, South Jeddah, Dahaban, Thuwal and Mastorah mangrove stands. However, the source of the biggest stress at Rabigh is salinity (r = 0.9012), as it influences antioxidants more than any other stressor. At the same time, the higher nutrient concentrations enhanced mangrove height even at high salinity. High temperature and low DO cannot be ruled out as sources of stress. The difference in δ^13^C enrichment across the mangrove stands or extra enrichment, especially at Rabigh relative to other mangrove ecosystems, was caused by stress resulting from extreme salinity, which confirms salinity to be the primary contributor of stress. The findings in this research suggest the use of nutrients and physicochemical parameters of seawater and antioxidant enzyme activities in mangroves can be used as proxies for oxidative stress and manage mangrove ecosystems across the Red Sea.

## Supporting information

S1 File Review cover letter. (DOCX)
