# Supplementary material for: Nutrient and physicochemical properties as potential causes of stress in mangroves of the central Red Sea
Source: PLoS One. 2021 Dec 23;16(12):e0261620. doi: 10.1371/journal.pone.0261620 (PMC8700010; doi:10.1371/journal.pone.0261620)
Supplement: S1 File — (DOCX) [file pone.0261620.s001.docx]

Editor-in-Chief

PLOS ONE

With the consent of all authors, I am submitting the final corrected version of our manuscript to **PLOS ONE** for possible publication. This is the only journal where the work has been submitted and has not been published elsewhere.

The necessary corrections were highlighted in red (Track Changes). We will also like to make correction to our funding information (grant number), the funding information (grant number) should be as stated below:

**Funding Information**: This research work was funded by Institutional Fund Projects under grant no. (**IFPIP-466-130-1442**). The authors gratefully acknowledge technical and financial support from the Ministry of Education and King Abdulaziz University, Jeddah, Saudi Arabia.

We will be looking forward to hearing from you soon.

Thank you.

**Corresponding author**:


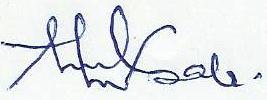


Abdullahi Bala Alhassan (Ph.D.)

Department of Biological Sciences, Faculty of Sciences, King Abdulaziz University, Jeddah, KSA.

+966541046505

[aalhassan0021@stu.kau.edu.sa](mailto:aalhassan0021@stu.kau.edu.sa)
